# A Fungal World: Could the Gut Mycobiome Be Involved in Neurological Disease?

**DOI:** 10.3389/fmicb.2018.03249

**Published:** 2019-01-09

**Authors:** Jessica D. Forbes, Charles N. Bernstein, Helen Tremlett, Gary Van Domselaar, Natalie C. Knox

**Affiliations:** ^1^Department of Internal Medicine, University of Manitoba, Winnipeg, MB, Canada; ^2^IBD Clinical and Research Centre, University of Manitoba, Winnipeg, MB, Canada; ^3^National Microbiology Laboratory, Public Health Agency of Canada, Winnipeg, MB, Canada; ^4^Centre for Brain Health and Faculty of Medicine (Neurology), University of British Columbia, Vancouver, BC, Canada; ^5^Department of Medical Microbiology and Infectious Diseases, University of Manitoba, Winnipeg, MB, Canada

**Keywords:** gut, mycobiome, mycobiota, fungi, neurological disease, multiple sclerosis

## Abstract

The human microbiome has received decades of attention from scientific and medical research communities. The human gastrointestinal tract is host to immense populations of microorganisms including bacteria, viruses, archaea, and fungi (the gut microbiota). High-throughput sequencing and computational advancements provide unprecedented ability to investigate the structure and function of microbial communities associated with the human body in health and disease. Most research to date has largely focused on elucidating the bacterial component of the human gut microbiota. Study of the gut “mycobiota,” which refers to the diverse array of fungal species, is a relatively new and rapidly progressing field. Though omnipresent, the number and abundance of fungi occupying the human gut is orders of magnitude smaller than that of bacteria. Recent insights however, have suggested that the gut mycobiota may be intricately linked to health and disease. Evaluation of the gut mycobiota has shown that not only are the fungal communities altered in disease, but they also play a role in maintaining intestinal homeostasis and influencing systemic immunity. In addition, it is now widely accepted that host-fungi and bacteria-fungi associations are critical to host health. While research of the gut mycobiota in health and disease is on the rise, little research has been performed in the context of neuroimmune and neurodegenerative conditions. Gut microbiota dysbiosis (specifically bacteria and archaea) have been reported in neurological diseases such as multiple sclerosis, amyotrophic lateral sclerosis, and Alzheimer's, among others. Given the widely accepted bacteria-fungi associations and paucity of mycobiota-specific studies in neurological disease, this review discusses the potential role fungi may play in multiple sclerosis and other neurological diseases. Herein, we provide an overview of recent advances in gut mycobiome research and discuss the plausible role of both intestinal and non-intestinal fungi in the context of neuroimmune and neurodegenerative conditions.

## Introduction

The human microbiome has entered into the forefront of scientific research, with growing importance for both the medical and research communities, although knowledge of its existence stems back decades (Eckburg et al., 2005). Commensal bacteria, particularly in the gut, are immensely beneficial to host health via influence of nutrient uptake, food metabolism, energy homeostasis, pathogen colonization resistance, epithelial barrier integrity, and the host immunological response (Shreiner et al., 2015). A stable equilibrium of the multitude of resident microorganisms also appears important to optimize health and maintain homeostasis, at least in the adult population. The gut microbiome and related dysbiosis has been extensively studied in prototypical gut-related conditions, such as inflammatory bowel disease (IBD), and recent research is beginning to elucidate the role of the gut microbiome in other diseases such as rheumatoid arthritis, diabetes, cancer, psoriasis, and neurological disorders (Forbes et al., 2016; Golombos et al., 2018; Wang et al., 2018). The human microbiome refers to all microbial communities (i.e., bacteria, archaea, viruses, and fungi) and their genomes. However, despite considerable evaluation of bacterial communities, much less is known regarding these other key microorganisms.

**Figure 1:**
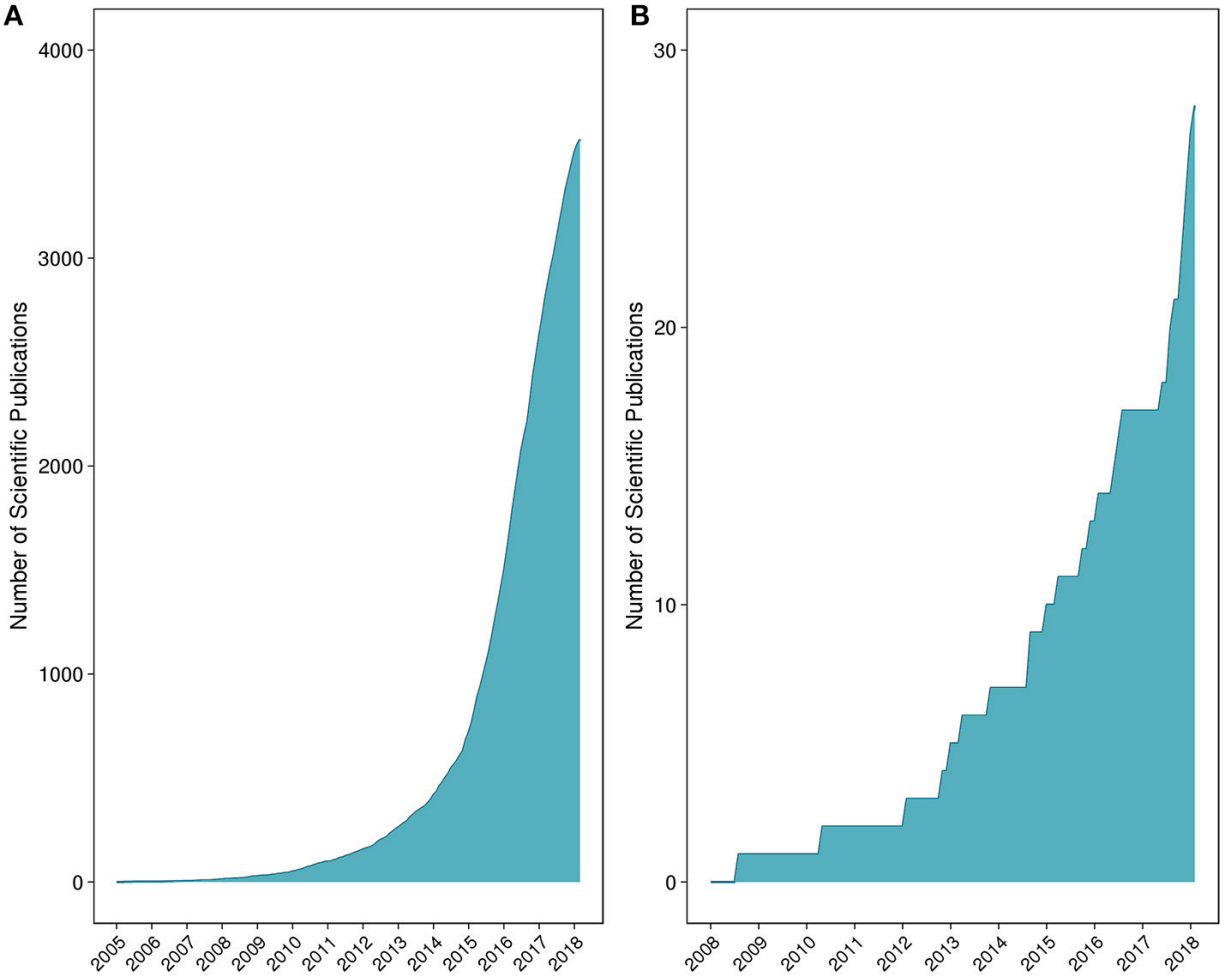
Number of peer-reviewed scientific publications for **(A)** microbiome and **(B)** mycobiome studies. The list of peer-reviewed literature was collated through iterative searches of the National Center for Biotechnology Information's PubMed database.

Fungi in particular represent an overlooked yet highly important kingdom. While studies have indicated that fungi are central to maintaining intestinal homeostasis and systemic immunity, <0.4% of the microbiome-related literature refer to or study fungal communities (Hernández-Santos and Klein, 2017). A schematic representation of the number of microbiome vs. mycobiome studies is provided in Figure 1. Research is beginning to reveal the importance of fungi on host health and also host-microbe and microbe-microbe interactions (Nash et al., 2017). Recent findings support the notion that a competitive association exists between bacterial and fungal microorganisms in the gut. As an example, studies have shown that prolonged antibiotic usage is linked to fungal infection and overgrowth, particularly in the gut, and that germ-free mice are susceptible to infection with fungi such as *Candida* (Noverr et al., 2004; Dollive et al., 2013). An antibiotic-induced fungal overgrowth in the mouse gut has also been shown to promote the development of allergic airway responses to *Aspergillus fumigatus* mold spores (Noverr et al., 2004). Moreover, commensal bacteria *Bacteroides thetaiotaomicron* and *Blautia producta* can induce the secretion of antifungal peptides via colonic epithelial cells (Fan et al., 2015). Comprehensive descriptions of bacteria-fungi and host-fungi interactions are reviewed elsewhere (Witherden and Moyes, 2018).

Study of the microorganisms that comprise the gut microbiome is of significant interest to the study of chronic diseases, particularly those with unknown causes or suboptimal therapeutic options. In this context, several links to the gut microbiome have been established such as in IBD, rheumatoid arthritis, psoriasis, ankylosing spondylitis, and systemic lupus erythematosus (Forbes et al., 2016). However, a causal association between the gut microbiome and most chronic diseases has yet to be established. Specifically in the context of neurological disorders, the most serious conditions are traditionally thought to affect the central nervous system (CNS). These conditions may have varying etiologies and pathobiologies such as chronic immune mediated inflammation in multiple sclerosis (MS) or chronic degeneration in amyotrophic lateral sclerosis (ALS) and Alzheimer's disease. Despite comprehensive clinical, experimental and biomedical research, the etiology of neurological disease is largely undefined and therapeutic interventions, such as drug treatments often remain far from ideal. Current thinking postulates that the cause of disease is multifactorial, modulated by a combination of genetic, environmental, and immunological influences (Korn, 2008). Numerous bacterial (i.e., *Mycobacterium tuberculosis, Neisseria meningitidis*), viral (i.e., human enteroviruses, herpes simplex virus-1), fungal (i.e., *Cryptococcus, Aspergillus*) and parasitic (i.e., *Trypanosoma cruzi, Toxoplasma gondii*) infections have the ability to affect the CNS with symptomatic presentation attributed to the infection or rather, the host immunological response^1^. Either directly or indirectly, the gut microbiome may have a role in the pathogenesis of a variety of neurological diseases. The complex relationship between the gut microbiome and the CNS has been termed the gut microbiota-brain axis (Dinan and Cryan, 2017). Herein, we explore the evidence surrounding the role of a relatively understudied group of microorganisms—fungi—and their potential to influence this microbiota-brain axis.

## Mycobiome Overview

Fungal constituents of the microbiome, termed the mycobiome, have received much less attention than other microorganisms, such as bacteria. Nonetheless, what is known about this emerging field and how the human mycobiome relates to health or disease is intriguing. Studies that have characterized the mycobiome in health have focused on various anatomical sites including the skin (Zhang et al., 2011), lungs (Nguyen et al., 2015), oral cavity (Ghannoum et al., 2010), and gastrointestinal tract (Huseyin et al., 2017b). The diversity of the human mycobiome has been shown to be quite variable between individuals and anatomical sites (Witherden and Moyes, 2018). These observations appear largely consistent with investigations of the bacterial microbiome (The Human Microbiome Project Consortium, 2012). To date, the *Ascomycota* phylum (including *Candida* spp., *Cladosporium* spp., and *Saccharomyces cerevisiae*) and the Basidiomycota phylum (including *Cryptococcus* spp., *Filobasidium* spp., and *Malassezzia* spp.) have been shown to dominate most human anatomic sites (Ghannoum et al., 2010; Zhang et al., 2011; Hoffmann et al., 2013; van Woerden et al., 2013).

## Methods to Study the Mycobiome

**Figure 2:**
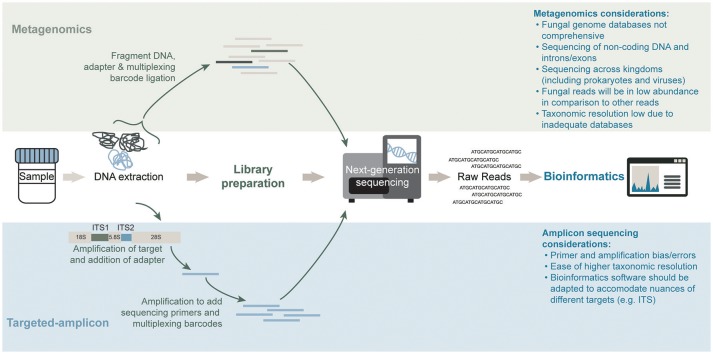
General workflows for mycobiota characterization of a shotgun metagenomics short-read sequencing approach (**top**) and targeted-amplicon sequencing of a phylogenetically informative fungal marker (**bottom**). Sequencing methods differ significantly in both approaches. The library preparation step in an amplicon sequencing approach requires an amplification step to generate the material for high-throughput sequencing whereas a metagenomics approach prepares the total genomic DNA content in a sample for high-throughput sequencing. Lastly, bioinformatics analysis of the raw reads for each method differs significantly in databases and software used.

Traditional methods for studying the mycobiome have included the use of culture-based methods. It has however been recognized that the inability to easily culture most fungal microorganisms of the microbiome are consistent with similar difficulties encountered for culturing the bacterial and archaeal members of the microbiome. To circumvent the challenges associated with culture-based methods, alternative molecular methodologies for studying the microbiome (and mycobiome) have been developed. One approach involves the use of a universal and taxonomically informative target, which can be amplified and sequenced using high-throughput sequencing (Figure 2). Through the use of reference sequence databases, target sequences can be taxonomically assigned and microbial abundances estimated for a profile of the community structure. Within the bacterial and archaeal kingdoms, the ubiquity of the 16S ribosomal RNA (rRNA) gene has functioned well for characterizing these microbial communities in diverse environments (Woese and Fox, 1977). Alternative targets for microbial community profiling include the 23S rRNA gene (Ludwig and Schleifer, 1994), chaperonin-60 universal target (*cpn*60 UT) (Links et al., 2012) and the β subunit of the bacterial RNA polymerase gene (rpoB; Mollet et al., 1997). However, due to vast genomic variations across all microorganisms (including prokaryotes, eukaryotes, and viruses) there is no universal target common to them all. In eukaryotes, the 18S and 28S rRNA genes, have been used to characterize the entire “microeukaryotic” community within an environment (Nash et al., 2017). Located between the 18S, 5.8S, and 28S rRNA genes are the internal transcribed spacers (ITS), named ITS1 (White et al., 1990) and ITS2 (Heisel et al., 2015). These regions are used to specifically target the fungal population (shown in Figure 2). Studies have reported differing mycobiome community profiles based on the selected ITS region indicating that ITS1 and ITS2 primers may present slight biases toward particular fungal species (Nash et al., 2017). Further, the high sequence and length variability of the ITS regions magnifies the complexity of the data analysis (Lindahl et al., 2013). The more conserved 18S rRNA target exhibits less overall bias when evaluating the mycobiome; however, its fungal taxonomic resolution is lower than that of the ITS regions (Schoch et al., 2012; Nash et al., 2017). An alternate untargeted approach is the use of shotgun metagenomics sequencing whereby a large number of short sequences are obtained from the random sampling of total extracted nucleic acids (Figure 2). Although ideal for its ability to sequence microorganisms across all kingdoms (and viruses), the sequencing depth required to accurately capture the less abundant microorganisms in a specimen can be difficult to attain, especially due to the presence of potentially overwhelming host and other microbial DNA, depending on the specimen (Hasan et al., 2016). Regardless of the molecular methodology used, a modified DNA extraction which favors the fungal community may be required due to difficulties with fungal cell lysis presumably associated with the presence of chitin in the cell wall (Dupuy et al., 2014; Vesty et al., 2017). A recent comparison by Nash et al. (2017) revealed that the use of an unmodified commercial extraction protocol yielded similar mycobiota profiles as the modified protocols which included harsher lysis methods.

Characterization of the mycobiome is complicated by the lack of comprehensive, well-curated, accurate, and high-resolution taxonomic annotation within fungal databases (Tang et al., 2015). Existing databases containing fungal targets include: UNITE (Abarenkov et al., 2010; Kõljalg et al., 2013), Findley (Findley et al., 2013), ITSoneDB (Santamaria et al., 2012), ITS2 database IV (Koetschan et al., 2012), RefSeq targeted loci [RTL; (Schoch et al., 2014)], targeted host-associated fungi (THF) database (Tang et al., 2015), International Society for Human and Animal Mycology (ISHAM) ITS database (Irinyi et al., 2015), and SILVA (Pruesse et al., 2007). The ribosomal database project (RDP) is also widely used for taxonomic classification purposes in fungal research (Cole et al., 2014). As the study of the mycobiome is in its early days and still rapidly evolving, computational tools and databases designed specifically for mycobiome analysis have yet to become widely established. As of September 2018, 3,520 fungal genome assemblies have been contributed to the National Center for Biotechnology Information (NCBI) assembly database compared to 162,834 bacterial genomes^2^. We anticipate that this number will rise dramatically in the coming years as more research is focused on investigating the involvement of fungi in health and disease. In comparison, the UNITE database currently contains over 817,130 ITS sequences^3^. Existing bioinformatics software originally used for the analysis of bacterial and archaeal communities have been adapted for mycobiota analyses. Such software includes, for community profiling, MEGAN (Huson et al., 2007), MetaPhlAn (Segata et al., 2012), QIIME (Caporaso et al., 2010), and mothur (Schloss et al., 2009). PICRUSt (Langille et al., 2013) can infer gene content directly from ITS2^4^ or 18S rRNA^5^. Alternatively, CloVR-ITS (White et al., 2013) and BROCC (BLAST read and operational taxonomic unit consensus classifier; (Dollive et al., 2013) software have been specifically developed to analyze sequence data for fungal taxonomic assignment.

In addition to database and analysis limitations, another issue affecting the potential accuracy of fungal species identification relates to the ability of some fungi to exist in two states (i.e., fungal dimorphism). The presence of fungi in either hyphal or yeast form has led to the misclassification of particular fungal microorganisms as being distinct despite being genetically identical thus propagating these erroneous taxa throughout public databases (Tang et al., 2015; Nash et al., 2017). Several studies have compared various methodological and analytical approaches of mycobiome assays (Dollive et al., 2012; Huseyin et al., 2017b; Nash et al., 2017). While significant advancements have been made in recent years with respect to bioinformatics methodologies for mycobiome analyses, further development of pipelines and databases will be necessary to more accurately characterize fungal communities.

## The Gastrointestinal Tract Mycobiome

A major focus of the literature on the human mycobiome has been to elucidate gut populations, which is unsurprising given the importance played by gut bacterial communities in health and disease. The gastrointestinal tract, which extends from the oral cavity to the anus, houses the largest and most diverse populations of microorganisms found within the human body (The Human Microbiome Project Consortium, 2012; Lloyd-Price et al., 2017). Several noteworthy studies and reviews in recent years have detailed the importance of fungi within the human gut (Hallen-Adams and Suhr, 2017; Huseyin et al., 2017b; Iliev and Leonardi, 2017; Li et al., 2017; Sam et al., 2017) and has highlighted the need for a more comprehensive gut mycobiome characterization. Examples of some key early studies in this area included investigations of the healthy gut (Dollive et al., 2012; Hoffmann et al., 2013) along with exploration in prototypical gastrointestinal diseases such as inflammatory bowel disease (IBD; Ott et al., 2008; Hoarau et al., 2016; Sokol et al., 2017).

It is widely recognized that the mycobiome exhibits less diversity and is present at much lower abundances relative to its bacterial counterpart (Qin et al., 2010). Ghannoum et al. (2010) reported the first sequencing-based approach to characterize the mycobiome of the healthy oral cavity of 20 individuals using a targeted-amplicon approach of the ITS regions. Among all oral rinse specimens, 74 culturable and 11 non-culturable genera were identified. Though some specimens were shown to exhibit substantial diversity (e.g., 39 or 16 genera) only 15 genera were observed in over 20% of specimens. The most frequently detected genera included *Candida, Cladosporium, Aureobasidium*, unidentified Saccharomycetales, *Aspergillus, Fusarium*, and *Cryptococcus*.

Similar to the bacterial microbiome, murine models have indicated that the highest concentrations of fungi are found in the distal colon (Iliev et al., 2012). Shotgun metagenomics sequencing approaches suggest that fungi account for approximately 0.1% of the gut microbiome (Qin et al., 2010) and an early study which included 96 stool samples from healthy volunteers found 66 genera (Hoffmann et al., 2013). However, this may be a significant underestimation attributed to challenges associated with genomic databases and the annotation of fungi, as already discussed.

Numerous fungi occupying the human gut have been reported, though few fungi have been shown to be common inhabitants across individuals or temporally. A recent review of 36 studies (published between 1917–2015) that employed either culture-dependent or –independent approaches reported that combined, 267 distinct species had been reported from the human gut (Suhr and Hallen-Adams, 2015). Overall, 15 species were reported in 5 or more studies, 37 species were reported in 2 or more studies, and 200 species were identified in a single study that employed culture-independent methodology and hence are likely non-culturable microorganisms. The authors reported that many species are observed in studies that incorporate several specimens, though interestingly, most of the detected taxa were present in only a single specimen. Taxonomic differences have also been observed to vary with sequencing methodology. Specifically, Dollive et al. (2012) investigated the fungal composition of stool from eight healthy donors via targeted-amplicon assays using either the 18S rRNA gene or ITS regions. For the 18S rRNA assay, most of the amplicon sequences were classified as *Saccharomyces* spp., whereas both *Saccharomyces* spp. and *Candida* spp. were frequently detected via the ITS assay. This discrepancy is largely due to the higher discriminatory power of the ITS region.

While most mycobiome research thus far has focused only on small cohorts, the Human Microbiome Project has recently surveyed the mycobiome of 317 healthy stool specimens using the ITS2 region, the 18S rRNA gene, and a shotgun metagenomics approach (Nash et al., 2017). Fungal diversity was shown to be significantly lower than bacterial diversity. Yeast was also shown to dominate the mycobiome; in particular, operational taxonomic units (OTUs) representing *Saccharomyces* (*S. cerevisiae*)*, Malassezia* (*M. restricta*), and *Candida* (*C. albicans*) were the most prevalent, present among 97, 88, and 81% of specimens, respectively. Considerable inter- and intra-individual variability was also observed. *S. cerevisiae, M. restricta*, and *C. albicans*, however, were detected in 92, 78, and 64% of individuals, respectively; these species are thought to be resident commensals and constitute a portion of the core mycobiome. The authors also reported that results from each approach were fairly consistent, with the exception of the ITS2 targeted-amplicon assay, which yielded greater resolution of low-abundance fungi. The notion of gut mycobiome instability presented in the above study (Nash et al., 2017) has been shown previously. Specifically, Hallen-Adams et al. (2015) reported that even potential core mycobiome members such as yeasts belonging to the Saccharomycetales or *Dipodascaceae* were rarely identified from the same individual in two specimens collected 3–4 months apart. Moreover, environmental fungi acquired through food products or airborne exposures comprise substantial OTU diversity, and these fungi often fail to colonize.

In a recent study, researchers set out to survey the resident gut fungi of 100 “healthy” volunteers involved in the Human Microbiome Project. Instead they concluded that all fungi identified (from stool) were likely transient and could be explained by dietary intake or fungi resident in the oral cavity (Auchtung et al., 2018). These findings support the notion that there is little to no fungal colonization in the otherwise “healthy” adult. The authors suggested that Westernization, evolution, ecology, and even the host immune response likely play a role in this phenomenon.

Despite increasing amounts of research on the gut mycobiome, a consensus healthy mycobiome has yet to be established. However, of the samples sequenced and published to date, the phyla Ascomycota, Basidiomycota, and to a lesser extent Zygomycota predominate, with more variability observed at lower taxonomic ranks. Fungal genera commonly detected in mycobiome assays include *Candida, Saccharomyces, Fusarium, Debaromyces, Penicillium, Galactomyces, Pichia, Cladosporium, Malassezia, Aspergillus, Cryptococcus, Trichosporon*, and *Cyberlindnera* (Hallen-Adams and Suhr, 2017; Wheeler et al., 2017). The potential roles played by these microorganisms in the human gut are described elsewhere (Hallen-Adams and Suhr, 2017).

### Factors That Affect the Gut Mycobiome

Some emerging factors thought to be associated with the community composition of the mycobiota include host genotype, host physiology such as sex, age, and presence of comorbid conditions, lifestyle such as diet, hygiene and occupation, and the immune system (Cui et al., 2013). Diet in particular represents a major factor influencing the fungal mycobiome composition (Huseyin et al., 2017a). Many fungi are foodborne microorganisms found in commonly consumed animal and plant-based food products and hence upon ingestion become transient colonizers that are thought to shape the gut mycobiome. In a study by Hoffmann et al. (2013), using unadjusted analyses via Spearman's correlation, the abundance of *Candida* spp. was shown to positively correlate with recent consumption of carbohydrates and negatively correlate with consumption of amino acids, proteins, and fatty acids. In the same study, *Aspergillus* spp. was shown to negatively correlate with recent consumption of short chain fatty acids (Hoffmann et al., 2013). Additional studies have shown *Candida* spp. and *Penicillium* spp. in stool to negatively correlate with almond and pistachio consumption (Ukhanova et al., 2014). Community differences have also been observed with broad diet groups such as vegetarian or Western diets (Hallen-Adams et al., 2015; Suhr et al., 2016), and between obese and non-obese individuals (Rodríguez et al., 2015). Combined, these studies support an association between diet and composition of the gut mycobiota; however, further research is needed to establish causation and explore the importance of additional factors that may influence the mycobiota, such as other lifestyle factors (e.g., exercise) or medications (e.g., antibiotics or antifungals) and comorbid conditions.

## Gut Mycobiome and Disease Susceptibility

Several studies have begun to investigate the relationship between the gut mycobiota and IBD (Hoarau et al., 2016; Sokol et al., 2017). Overall, the gut mycobiome of IBD patients are characterized by reduced fungal diversity and a dysbiosis in community populations relative to healthy controls (Hoarau et al., 2016; Sokol et al., 2017). At the phylum level in particular, the ratio of Basidiomycota to Ascomycota is altered. There is also a significantly higher relative abundance of Basidiomycota and a corresponding lower abundance of Ascomycota. More specifically, these trends are mostly attributed to a higher relative abundance of the taxa *Candida, Filobasidiaceae*, and Malasseziales and a concurrent lower abundance of *Saccharomyces, Penicillium*, and *Kluyveromyces*. While similar observations have been reported across studies (e.g., lower *S. cerevisiae* and higher *Candida* spp.), differences have been reported particularly at the species level (Hoarau et al., 2016; Sokol et al., 2017). For example, Hoarau et al. (2016) described higher abundances of *C. tropicalis* whereas Sokol et al. (2017) reported higher *C. albicans*, though it is possible these discrepancies may be attributed to differences in sequencing targets (i.e., ITS1 vs. ITS2). Of importance, *Candida* spp. have been implicated in numerous disorders such as IBD and even neurological disease (Sánchez–Portocarrero et al., 2000; Ott et al., 2008).

### Gut Mycobiome in Neurological Disease

With emerging evidence that the gut microbiome is intricately involved in neurological disease (Forbes et al., 2016; Tremlett et al., 2017), it is reasonable to speculate that the fungal component plays an important role along with other members of the gut microbiome (i.e., bacteria and archaea). Most research exploring a fungal association to date (discussed below) has focussed on MS, and relatively little is known regarding other neurological diseases (Table 1). MS is an immune-mediated inflammatory disease that shares overlapping epidemiological characteristics (e.g., demographics, geographic location) and etiopathophysiology (e.g., risk factors, genetic susceptibility, immunological aberrancies) with IBD (Lin et al., 2014; Kosmidou et al., 2017) and other chronic immune diseases. A recent review also described differences in bacterial abundances that are common to both IBD and MS (Bernstein and Forbes, 2017). Based on these observations, it is conceivable that the mycobiome, which has been shown to be perturbed in IBD (Hoarau et al., 2016; Sokol et al., 2017), is similarly altered in neurological disease.

**Table 1:** Key findings related to the gut mycobiome in neurological disease.

Link between the CNS and the gut microbiome is not established
Presence of fungi in the gut may be underreported due to methodologies (wet-laboratory and bioinformatics)
What is known about the gut mycobiome in human health
Fungal colonization in the gut is thought to be limited
The “core” mycobiome is suggested to be small
High diversity within (temporal) and across individuals
Fungi may act as an opportunistic pathogen reservoir
Some fungal species may have a beneficial effect
Probiotic effect reported for some fungi
Re-myelinating potential shown to induce remission or prevention of relapse
Fungal mycobiome studies in neurological disease is largely focused on MS with limited research in others
Implication of fungi in MS
Increased prevalence of fungal infections in blood and CSF of MS patients
Elevated presence of fungal antibodies in neurological specimens
Fungal infections in blood and neurological specimens may be a risk factor for MS
Fungal toxin produced by pathogenic fungi in the gut may cross blood-brain barrier and play a role in myelin degradation
Implication of fungi in Alzheimer's disease and ALS
Evidence of fungi in neurological specimens
Where more studies are needed in neurological conditions
Role of fungi in neurological and gastrointestinal specimens
Shotgun metagenomics studies to characterize the mycobiome in various body sites
Characterization of host-fungi and fungi-bacteria interactions

Increasing interest of the mycobiome in neurological disease is largely driven by findings that specific fungi can modulate the host immunological response and hence may be a risk factor for immune diseases in genetically susceptible hosts (Dworecka-Kaszak et al., 2016). Recent data provide supportive evidence that the mycobiota-immunity link affects both the local and systemic immune response (Wheeler et al., 2016). The mycobiome may also function as a reservoir for opportunistic pathogens in immunocompromised individuals, hence playing a role in diseases not obviously linked to the gut. Specifically, fungi have previously been shown to act as opportunistic pathogens during immune-mediated and antibiotic therapy (Gutwinski et al., 2010; Penkert et al., 2016; Seto et al., 2016). In this context, fungal infections represent a large proportion of infectious diseases in immunocompromised persons (Drgona et al., 2014). In contrast, potential gut health benefits or probiotic effects of some fungal species such as *Saccharomyces boulardii* (Ward et al., 2017) are also being explored. Thus, there is emerging supportive evidence to suggest a potential association between the gut mycobiome and neurological disease; however, at present (March 2018) no study has attempted to characterize the gut mycobiome in neuroimmune or neurodegenerative disorders. Interestingly, perturbed gut mycobiotas have been reported in neurodevelopmental conditions such as autism spectrum disorder (Strati et al., 2017) and Rett syndrome (Strati et al., 2016).

While gut mycobiome research in neurological disease is lacking, several studies investigating the role of fungi in neurological disease have taken the approach of searching for a fungal association using biological specimens such as serum, CSF or neural tissue. These fungal associations have generally been in the form of identifying fungal infections whereby non-commensal microorganisms cause acute illness, or alternatively, the overgrowth of commensal fungi.

### Multiple Sclerosis

As the prototypical neurological inflammatory disease, much investigation has been aimed to elucidate the etiopathogenic mechanisms of MS. Many infectious microbial agents, particularly viruses (e.g., Epstein Barr virus) and some bacteria have been investigated as potential causes of MS, with varying degrees of evidence (McKay et al., 2016). Recent studies have also suggested that archaea or fungi may play a role (Tremlett et al., 2017). The association between infections and the development of MS has been suggested by the identification of antibodies in serum and cerebrospinal fluid (CSF) and by the detection of proteins or nucleic acid sequences in CSF that are diagnostic for a given pathogen. Many mechanisms exist whereby an infectious agent might trigger an autoreactive host immunological response such as molecular mimicry, epitope spreading, and bystander activation (Dendrou et al., 2015). Experimental evidence supporting a putative link between fungi and MS has recently been reviewed elsewhere (Benito-León and Laurence, 2017). Specifically, host genetic susceptibility (i.e., HLA-DRB1^*^15 allele group), biomarkers such as IL-17, chitotriosidase, and antibodies against fungi and the administration of the disease-modifying drug, dimethyl fumarate, for the treatment of MS (which, incidentally, can suppress molds) collectively suggest there could be a fungal etiology of MS (Benito-León and Laurence, 2017).

In terms of fungal overgrowth or infection in MS, most evidence thus far has explored the association between disease and *Candida* spp. Symptomatic improvement of five MS patients following antifungal therapy was reported in an uncontrolled study, with no comparator group (Truss, 1981). Of relevance, however, these results have not been replicated. Ramos et al. (2008) reported the presence of fungal infection in seven MS patients with elevated antibody titers against *Candida* spp., fungal DNA in the blood of six of these patients and β-1,3 glucan in the serum of four patients. Results were also compared to 10 healthy controls. Antibodies and antigens to yeast were also found in CSF. The authors proposed that MS could be caused by fungal infection or that an aberrant host immunological response influences fungal proliferation. In a larger case-control study including 80 MS patients and 240 matched controls, Benito-León et al. (2010) determined that a *Candida* infection might be serologically linked to MS. Blood specimens were subjected to immunofluorescence analysis (IFA) and enzyme-linked immunosorbent assay (ELISA) to detect *Candida* antibodies and slot-blot for the detection of antigens. Higher concentrations of serum antibodies were reported among MS patients relative to controls. Specifically, *Candida famata* was reported in 37.5% of MS patients vs. 12.5% of controls (*p* < 0.001), *C. albicans* in 47.5% vs. 21.3% (*p* < 0.001), *C. parapsilosis* in 37% vs. 17.1% (*p* < 0.001), and *C. glabrata* in 46.3% vs. 17.5% (*p* < 0.001). A higher odds of MS was associated with the presence of *Candida* antigens including *C. parapsilosis* (OR = 7.3, 95% CI 3.2–16.6, *p* < 0.001) and *C. glabrata* (OR = 3.0, 95% CI 1.5–6.1, *p* = 0.002).

In a longitudinal study of one person with MS over 3 years, serum levels of antibodies against different *Candida* spp. fluctuated. However, the pathological implications of antibodies to Candida are unknown nor has this been studied in a larger cohort. Several fungal species within serum samples were detected via PCR and sequencing. Antibodies against *Candida* spp. and antigens related to *C. famata* were similarly found in CSF. Further research by this group analyzed both blood and CSF specimens from 12 new-onset MS patients with “minimal disability” (i.e., a score of 1 on the Expanded Disability Status Scale, EDSS) and reported evidence of fungal macromolecules (proteins and DNA) in the CSF of some patients (Pisa et al., 2013). While assays performed in this study such as IFA, ELISA, slot-blot, and PCR prove to be useful for the detection of fungal antigens and antibodies reacting to *Candida* spp., the authors have shown that concomitant analysis of blood and CSF may be an effective method for the determination of disseminated fungal infection. The authors reported that the presence of a fungal infection may be a risk factor for disease and may also aid in understanding the etiopathogenesis of MS. Of note, the original source of the infection or fungi remains unclear in each of the studies mentioned.

Other research has investigated the role of *C. albicans* via the specific activity of proteinase A in the severity of MS (Saroukolaei et al., 2016). *C. albicans* strains were isolated from superficial surfaces of MS patients and healthy controls. Analytical models which considered both specific enzyme activity (SEA) and EDSS were applied. The SEA of *C. albicans* in MS was significantly higher than controls and although positive correlations with EDSS were observed (*p* < 0.001, *r* = 0.65), similar correlation coefficients were found when EDSS was replaced with either disease duration or age (Saroukolaei et al., 2016).

It has been proposed that fungal toxins may play a role in the destruction of astrocytes and oligodendrocytes thereafter leading to characteristic myelin degradation (Benito-León et al., 2010; Purzycki and Shain, 2010). Purzycki and Shain (2010) theorized that particular pathogenic fungi such as *Aspergillus* and *Candida* spp., which are masked from the immune system by their mannan coats, are housed in non-neuronal tissue (e.g., gastrointestinal tract) and gradually release toxins such as gliotoxin into the bloodstream. Once toxins cross the blood-brain barrier, CNS astrocytes, which are integral in maintaining barrier integrity and oligodendrocytes, which provide nutritional maintenance for myelin, are targeted. The blood-brain barrier therefore weakens, myelin degrades and the symptomatic MS presentation occurs with specific disease courses driven by distinct mycotoxins. To date, however, this remains speculative as supportive evidence is lacking.

Few metagenomics assays have been performed using CSF from MS patients; in each study, cross- or environmental contamination was thought to be an issue. Perlejewski et al. (2016) examined CSF from 12 patients with idiopathic inflammatory demyelinating disorders, including 10 MS patients, and found that fungal in addition to bacterial, parasitic, and protozoan reads were identified in all samples at comparable levels, indicative of a common presence of contamination. Jovel et al. (2017) recently performed a metagenomics sequencing assay on CSF from 28 MS patients and 15 patients with other neurological conditions. While the presence of bacterial reads was mostly thought to be related to contamination, viral reads corresponding to Epstein-Barr virus, and cytomegalovirus were detected. There was however no mention of fungal reads identified in any specimen. The authors concluded that the CSF of MS patients could be considered largely free of microbial DNA.

### Other Neurological Disorders

The possible role of fungi has similarly been explored in other neurological disorders such as Alzheimer's disease and amyotrophic lateral sclerosis (ALS), however, scientific evidence is lacking for other neurological conditions. Alzheimer's disease is a condition characterized by the presence of amyloid plaques in the brain, neurofibrilliary tangles that influence neuronal cell death, vascular dysfunction, and inflammation. Alonso and colleagues have published a series of 4 reports elucidating a possible link between fungi and Alzheimer's disease (Alonso et al., 2014a,b, 2017a; Pisa et al., 2015). In a cohort of 29 Alzheimer's disease patients, a battery of assays were conducted to investigate the prevalence of fungal infections from blood serum (Alonso et al., 2014b). These assays included the detection of antibodies against *Candida* spp. (e.g., *C. famata, C. albicans, C. parapsilosis, C. glabrata, C. krusei*) by immunofluorescence assay, detection of antigens from *Candida* spp. and other fungal species (e.g., *Saccharomyces cerevisiae, Rhodotorula mucilaginosa*) and the presence of fungal polysaccharides (e.g., β-1,3 glucan) via the Fungitell assay. In analysis of anti-*Candida* antibodies, patient-related variability was observed. While immunoreactions were reported and measurable in the majority of patients, Alzheimer's disease patients exhibited high, intermediate, or low reactivity. Slot-blot technique of fungal species revealed that very high levels of fungal antigens were detected in numerous Alzheimer's disease patients. Fungal polysaccharides were detected in the majority of Alzheimer's disease patients (values above 80 using the Fungitell assay). The authors also compared Alzheimer's disease patients to 60 healthy blood donors and reported; significant differences (*p* < 0.05) were reported for *C. famata, C. albicans*, and *C. glabrata*. The authors also detected fungi in brain specimens by combining proteomics and genomics (e.g., sequencing and PCR) approaches (Alonso et al., 2014a). Proteomics analysis suggested fungal proteins in the brain of Alzheimer's disease patients while sequencing revealed that fungal species could be found in the brain from Alzheimer's disease patients. PCR revealed numerous fungal species, which was determined to be dependent on the patient and tissue. In a separate study, Pisa et al. (2015) reported fungal species to vary across 14 Alzheimer's disease patients and by brain regions which was also confirmed by sequencing of fungal DNA extracted from frozen CNS specimens. The sequencing approach amplified both ITS1 and ITS2. The external frontal cortex, cerebellar hemisphere, entorhinal cortex/hippocampus and choroid plexus contained fungal cells and/or hyphae, which were largely absent in 11 brain specimen controls. Of note, no single fungal species was identified in all 4 regions examined of the CNS. *Malasezzi* spp., *Phoma*, and *S. cerevisiae* were identified in the CNS of Alzheimer's disease patients. Subsequent work from the same group employed a high-throughput targeted-amplicon sequencing approach of 4 distinct brain regions of one patient with Alzheimer's disease (Alonso et al., 2017a); *Botrytis cinerea* and *Cryptococcus curvatus* were reported in all regions. Further, entorhinal/cortex hippocampus specimens from an additional 8 patients revealed numerous fungal species. Notably, 5 genera including *Alternaria, Botrytis, Candida, Cladosporium*, and *Malassezia* were common to all patients. Despite limitations of this study (i.e., small number of cases and controls and lack of replication by an independent cohort), the authors concluded that these findings could guide future targeted antifungal therapy for patients with Alzheimer's disease.

The same authors explored a fungal association in amyotrophic lateral sclerosis (ALS), a common neurodegenerative disorder characterized by progressive motor neuron dysfunction of the motor cortex, brainstem, and spinal cord. Alonso et al. (2015) detected fungal antigens and DNA corresponding to numerous fungi from CSF in 5 ALS patients compared to 3 controls. The average age of ALS patients was 70.8 (2 male; 3 female) whereas the average age of healthy donors was 58.3 (2 male; 1 female). In the same study, immunopositive fungal antigens (i.e., punctate bodies) were also observed in the frontal cortex of six ALS patients, compared to 4 healthy donors. Fungal DNA was similarly detected in brain tissue via a PCR approach, which corresponded to several fungal species. *Malassezia globosa* was observed in most samples and *Cryptococcus neoformans* was detected in a single sample. *C. albicans* was also commonly detected. In addition, mass spectrometry analyses revealed the occurrence of numerous (e.g., >2,000) fungal peptides though only 4 could be defined as certain fungal peptides. A subsequent study of brain specimens from 11 ALS patients using immunohistochemistry identified fungal structures (i.e., yeast and hyphae) in the spinal cord, medulla and motor cortex (Alonso et al., 2017b). These observations were not found among the 4 controls. High-throughput targeted amplicon sequencing and analysis identified numerous fungal species such as *Candida, Malassezia, Fusarium, Botrytis, Trichoderma*, and *Cryptococcus*. Though preliminary, the authors suggested that these findings provide evidence for mixed fungal infections among ALS patients. A recent report, published as a conference abstract, applied a metagenomics sequencing approach on isolated RNA from lumbar motor neurons of 11 ALS patients and 8 controls in addition to DNA from the frontal cortex of 209 persons with Alzheimer's disease and included 192 control neurons from NCBI's Sequence Read Archive (SRA) for a comparative study (Keith and Mitchell, 2017). Though no particular fungi were described, fungi were reported to represent 0.0004 and 0.001% of metagenomics sequences, for Alzheimer's disease and ALS, respectively.

## Gut Mycobiome Therapeutic Manipulation

Gut microbiome manipulation with antibiotics, prebiotics, or probiotics is of particular interest in the context of improving health outcomes, although deleterious effects are also a major health concern. Exposure to broad-spectrum antibiotics, for example, is a well-known risk factor for the development of some conditions, such as *Clostridium difficile* infections (Lamendella et al., 2018). However, some antibiotics are also a treatment for the condition. Indeed, there is potential for any of these interventions to impact disease course by altering the composition of gut bacteria, which may result in, for instance, the induction of remission or prevention of relapse. Manipulation of the gut mycobiota to elicit a therapeutic response is similarly intriguing. The mycobiome has recently been recognized and granted a patent for its potential as a probiotic, diagnostic and/or treatment tool, specifically in ulcerative colitis (Underhill and Iliev, 2016). Moreover, using a mouse model of MS, miconazole was recently shown to function as a remyelinating drug with no measurable adverse effects on the host immune system (Najm et al., 2015). Miconazole (as well as clobetasol, a corticosteroid) was shown to enhance generation of human oligodendrocyte progenitor cells *in vitro*. This proof-of-principle study provides scientific rationale to test miconazole or clobetasol (or related compounds) as promoters of remyelination in MS patients. This ability to promote repair represents a major unmet need in MS; currently, there are no drugs approved for MS which target remyelination (Plemel et al., 2017). Though it does not necessarily establish a link between targeting the mycobiome and MS therapy, it is intriguing that these antifungal/anti-inflammatory compounds do have a therapeutic benefit in MS.

Fecal microbiota transplantation (FMT) is an approach increasingly being evaluated to treat diseases linked to gut dysbiosis. There are a few case reports, which suggest the potential benefit of FMT in neurological diseases (Borody et al., 2011, 2012; Makkawi, 2017) and further, clinical trials are currently underway to investigate FMT in relapsing MS patients. While the premise behind FMT is the introduction of a stable gut microbial community following the transfer of healthy donor stool, studies have mostly evaluated the bacterial community shifts. It would be useful to understand how the mycobiota shifts following FMT and whether this conveys a health benefit.

## Conclusions and Perspectives

The study of the mycobiome in human health and disease is clearly still in its infancy. Significant advancements in high-throughput sequencing and computational capacity have made it increasingly possible to evaluate the mycobiome. Although improvements in wet-laboratory techniques and bioinformatics tools are ongoing, this promising field will likely evolve considerably in the coming years. In particular, as capacity to undertake complex metagenomics and targeted-amplicon studies increases, so will the ability to elucidate the potential importance of fungi in human health. While we have discussed studies whereby the presence of a fungal infection and/or metagenomics sequencing assays were conducted to investigate fungal involvement in neurological disease, there is a clear gap in the scientific literature. We advocate that future research investigate the human mycobiome in neurological disease using biological specimens including serum or CSF, employing metagenomics approaches. Studies are similarly needed to investigate the gut mycobiome involvement in neurological disease (i.e., stool specimens). Further, gut mycobiome studies are needed to explain the nature of the relationship and better understand any potential confounding factors or effect modifiers, such as age, sex, diet, comorbid conditions, or geographical location. There is a need for continued refinement of several aspects of methodological studies, such as sequenced isolates, and improvements to reference databases and annotation. Additional research is required to understand the likely complex and multifaceted interactions between fungi and host and between fungi and other groups of microorganisms. Therapeutic manipulation of the gut microbiome holds promise as a treatment of chronic diseases; how and if the mycobiome might contribute remains to be determined. Future research should be performed to further investigate the role of targeted and untargeted interventions, such as antifungals and FMT in neurological disease. We are only beginning to understand the potential structure, role and functions of the gut mycobiome and its ability to maintain health and cause or propagate disease. At present, fungi represent an overlooked kingdom of the microbiome and may also represent an underappreciated component of neurological disease.

## Author Contributions

JF and HT developed the concept for the manuscript. JF and NK wrote the manuscript. All authors reviewed, edited, and approved the final version of the manuscript.

### Conflict of Interest Statement

HT is the Canada Research Chair for Neuroepidemiology and Multiple Sclerosis. She currently receives research support from the National Multiple Sclerosis Society, the Canadian Institutes of Health Research, the Multiple Sclerosis Society of Canada and the Multiple Sclerosis Scientific Research Foundation. In addition, in the last 5 years she has received research support from the Multiple Sclerosis Society of Canada (Don Paty Career Development Award); the Michael Smith Foundation for Health Research (Scholar Award) and the UK MS Trust; speaker honoraria and/or travel expenses to attend conferences from the Consortium of MS Centers (2013), the National MS Society (2014, 2016), ECTRIMS (2013, 2014, 2015, 2016, 2017), Biogen Idec (2014), American Academy of Neurology (2013, 2014, 2015, 2016). All speaker honoraria are either declined or donated to an MS charity or to an unrestricted grant for use by her research group. CB is supported in part by the Bingham Chair in Gastroenterology. He has served on advisory boards of Abbvie Canada, Ferring Canada, Janssen Canada, Pfizer Canada, Shire Canada, Takeda Canada, Napo Pharmaceuticals and has consulted to 4D Pharm and Mylan Pharmaceuticals. He has received educational grants from Abbvie Canada, Janssen Canada, Pfizer Canada, Shire Canada and Takeda Canada. GV receives operational funding from the Public Health Agency of Canada and has also received research support from the Multiple Sclerosis Society of Canada, the Canadian Institutes of Health Research, The National Sciences and Engineering Research Council, and Genome Canada. The remaining authors declare that the research was conducted in the absence of any commercial or financial relationships that could be construed as a potential conflict of interest.

## Footnotes

^1^http://www.who.int/features/qa/55/en/

^2^https://www.ncbi.nlm.nih.gov/assembly/

^3^https://unite.ut.ee/

^4^https://github.com/LangilleLab/microbiome_helper/wiki/ITS2-Fungi-Standard-Operating-Procedure

^5^https://github.com/LangilleLab/microbiome_helper/wiki/18S-Eukarya-Standard-Operating-Procedure

## Abbreviations

ALS: amyotrophic lateral sclerosis
BROCC: BLAST read and operational taxonomic unit consensus classifier
CNS: central nervous system
CSF: cerebrospinal fluid
DNA: deoxyribonucleic acid
EDSS: expanded disability status scale
ELISA: enzyme-linked immunosorbent assay
FMT: fecal microbiota transplantation
IBD: inflammatory bowel disease
IFA: immunofluorescence analysis
ISHAM: international society for human and animal mycology
ITS: internal transcribed spacer
MS: multiple sclerosis
NCBI: National Center for Biotechnology Information
PCR: polymerase chain reaction
RNA: ribonucleic acid
rRNA: ribosomal ribonucleic acid
RTL: RefSeq targeted loci
SEA: specific enzyme activity
SRA: sequence read archive
THF: targeted host-associated fungi.
